# Evaluation of sleep quality and influencing factors among medical and non-medical students using machine learning techniques in Fujian during the public health emergencies

**DOI:** 10.3389/fpsyt.2025.1533875

**Published:** 2025-06-06

**Authors:** Yifei Lin, Qingquan Chen, Zeshun Chen, Shengxun Qiu, Liangming Wang

**Affiliations:** ^1^ The Second Affiliated Hospital of Fujian Medical University, Quanzhou, Fujian, China; ^2^ The School of Clinical Medicine, Fujian Medical University, Fuzhou, Fujian, China; ^3^ The School of Public Health, Fujian Medical University, Fuzhou, Fujian, China

**Keywords:** medical students, non-medical students, COVID-19, sleep quality, machine learning

## Abstract

**Background:**

The COVID-19 pandemic has significantly affected the sleep quality of medical and non-medical students, yet the influencing factors remain unclear. Objective: This study aimed to assess sleep quality of 20,645 full-time undergraduate and graduate students aged between 17–35 years old in Fujian Province who were enrolled in universities and colleges in the province and to explore key influencing factors while establishing predictive models.

**Methods:**

A cross-sectional survey was conducted using an online questionnaire from April 5 to 16, 2022, employing demographic survey components, coffee use, internet use, psychological factors and the Pittsburgh Sleep Quality Index (PSQI). Data were analyzed with a training set (70%) and testing set (30%), utilizing four machine learning techniques: naive Bayes, artificial neural networks, decision trees, and gradient boosting trees.

**Results:**

Non-medical students exhibited poorer sleep quality than medical students (P<0.001). Risk factors for non-medical students included age ≥20 years and fear of infection, while graduation class was a determinant for medical students. The developed models demonstrated high clinical efficiency, with strong agreement between predictions and observations, as shown by calibration curves. Decision curve analysis indicated net benefits for all models.

**Conclusions:**

Non-medical students faced more factors affecting their sleep quality. The validated prediction models provide accurate estimations of sleep disorders in college students, offering valuable insights for campus management.

## 1 Introduction

The issue of sleep quality has not only garnered significant attention in China but has also become a focal point of global concern. Inadequate sleep not only disrupts the physiological rhythm of the body, compromising the immune system, but it also detrimentally affects cognitive abilities (1). Prolonged sleep deprivation can lead to sleep disorders, heighten the risk of infectious diseases, and contribute to the onset and progression of various ailments, including depression (2–4).

Since the declaration of COVID-19 as a global public health emergency by the World Health Organization (WHO) (5), China has implemented extensive, stringent, and comprehensive containment measures. These measures include family isolation, the transition to online learning, and the prohibition of social gatherings, all aimed at curbing the spread of the virus and minimizing the risk of human-to-human transmission. However, enforced self-isolation may have repercussions on individuals’ physical and mental health, consequently exerting a pronounced negative impact on healthy lifestyle behaviors (6).Furthermore, the intimate connection between sleep quality and the physical and mental well-being of young people is well-established (7). The prolonged absence of interventions addressing the physical and mental health of college students is likely to have enduring effects on their sleep quality.

In conclusion, the issue of sleep quality is multifaceted, encompassing physiological, cognitive, and mental health aspects. The additional stressors introduced by the COVID-19 pandemic and the associated containment measures underscore the importance of holistic approaches to health. Initiating strategies to support individuals, particularly vulnerable populations like college students, is crucial for mitigating the long-term impact on sleep quality and overall health. There exists a substantial body of literature investigating the sleep quality of university students during major public health emergencies such as the COVID-19 pandemic (8, 9). Additionally, several meta-analyses have examined the sleep quality of medical students (10, 11). However, scant attention has been given in the literature to assessing the impact of such events on the sleep quality of both medical and non-medical students. Furthermore, many studies have failed to develop predictive models for sleep quality among these student groups, which are crucial for providing effective guidance during future public health emergencies.Given these gaps, our study focuses on comparing the sleep quality between medical and non-medical students and on developing predictive models for both groups.

While medical students generally possess more profound medical knowledge and professional skills than their non-medical counterparts (12), they are also confronted with unique challenges, particularly during epidemics such as prolonged quarantines. Senior medical students, engaged in hospital internships and having increased patient contact, face an elevated risk of infection. This heightened exposure not only raises the likelihood of physical and mental health issues but also influences their sleep quality (13, 14). Numerous studies indicate that medical students are more susceptible to sleep disorders compared to non-medical students. Contributing factors include extended study hours, elevated intensity, clinical probation, overnight duties, emotional stress, and lifestyle choices, among others (15, 16).

Fujian Province, located on the southeast coast of China, is a region with a long history and rich cultural heritage. Fujian has a significant role in China’s economic development, with a strong focus on industries such as manufacturing, trade, and tourism. As a developed southeastern coastal province with significant population mobility and frequent international exchanges, Fujian Province is at a heightened risk for public health emergencies. This results in considerable pressure on prevention and control efforts, particularly regarding infectious diseases. The province’s multicultural environment may also influence students’ health perceptions and sleep habits. This study focuses on Fujian Province, controlling for variables to precisely analyze the impact of professional background on sleep quality. The educational backgrounds and living environments of medical and non-medical students in Fujian are relatively homogeneous, which helps mitigate the influence of external factors. Additionally, Fujian Province is frequently used as a pilot region for public health policies, and the insights gained here could serve as valuable references for other provinces.

Despite a substantial number of students reporting sleep-related problems (17), many educational institutions, including medical schools, lack comprehensive guidance and education on sleep and sleep disorders (18). Previous research has revealed that non-medical college students in Shandong Province, China, exhibit more mental health problems and risk factors than their medical counterparts during recurrent COVID-19 epidemics (19). However, there is a dearth of similar studies exploring the situation in Fujian Province, China. In conclusion, the distinctive challenges faced by senior medical students, particularly in the context of epidemic responses, highlight the need for targeted interventions to address their heightened risk of physical and mental health issues, including sleep disorders. Comprehensive education on sleep-related topics is essential across all academic disciplines, ensuring the well-being of students. Further research, specifically in regions such as Fujian Province, will contribute valuable insights into the unique challenges faced by students during epidemics, aiding in the development of tailored support mechanisms.

In recent years, machine learning (ML) has found widespread applications in the medical field, particularly in sleep medicine, where it exhibits significant potential. For instance, a study leveraged random forest technology to achieve a notable improvement in the accuracy of sleep stage classification, reaching an impressive 95.78% (20). Similarly, another study utilized machine learning to predict the sleep quality of college students, yielding promising results (8). Previous research has also highlighted the impact of epidemics on the sleep quality of Chinese citizens (21). However, there is a notable absence of literature focusing on the development and validation of prediction models for assessing the sleep quality of both medical and non-medical students.

Consequently, we conducted a questionnaire-based cross-sectional study to comprehensively assess the sleep quality of both medical and non-medical students in Fujian Province during the recurrent epidemic. This study aims to evaluate disparities in sleep quality between these two student groups in Fujian Province during epidemic events. Furthermore, it seeks to develop and validate prediction models, providing tailored support and recommendations to enhance the sleep quality of both medical and non-medical students during epidemics. This, in turn, contributes to help understand and control the public health emergencies.

## 2 Methods

### 2.1 Data sources

An Internet-based cross-sectional survey utilizing a simple random sampling method was conducted in Fujian Province, China to assess the sleep quality of both medical and non-medical students during the COVID-19 epidemic. In this study, questionnaires were collected from April 5 to 16; The period of closed management of universities was from March to April 2022. For the online survey, it was calculated as 22074 responses out of 1023362 students, resulting in a response rate of 2.16%.

The survey’s objectives and the principles of informed consent were comprehensively explained within the questionnaire content, ensuring respondent anonymity. All items were designated as mandatory to uphold questionnaire integrity. Each ID number or WeChat account was restricted to a single questionnaire submission to prevent duplicate responses. The inclusion criteria encompassed full-time undergraduate and graduate students in Fujian Province, China, aged between 17 and 35, enrolled in universities and colleges within the province. Exclusion criteria comprised: 1. Full-time professional students, university undergraduates, and graduate students residing in non-school isolated locations during the survey period; 2. Individuals with severe sleep disorders or major mental disorders.

**Figure 1:**
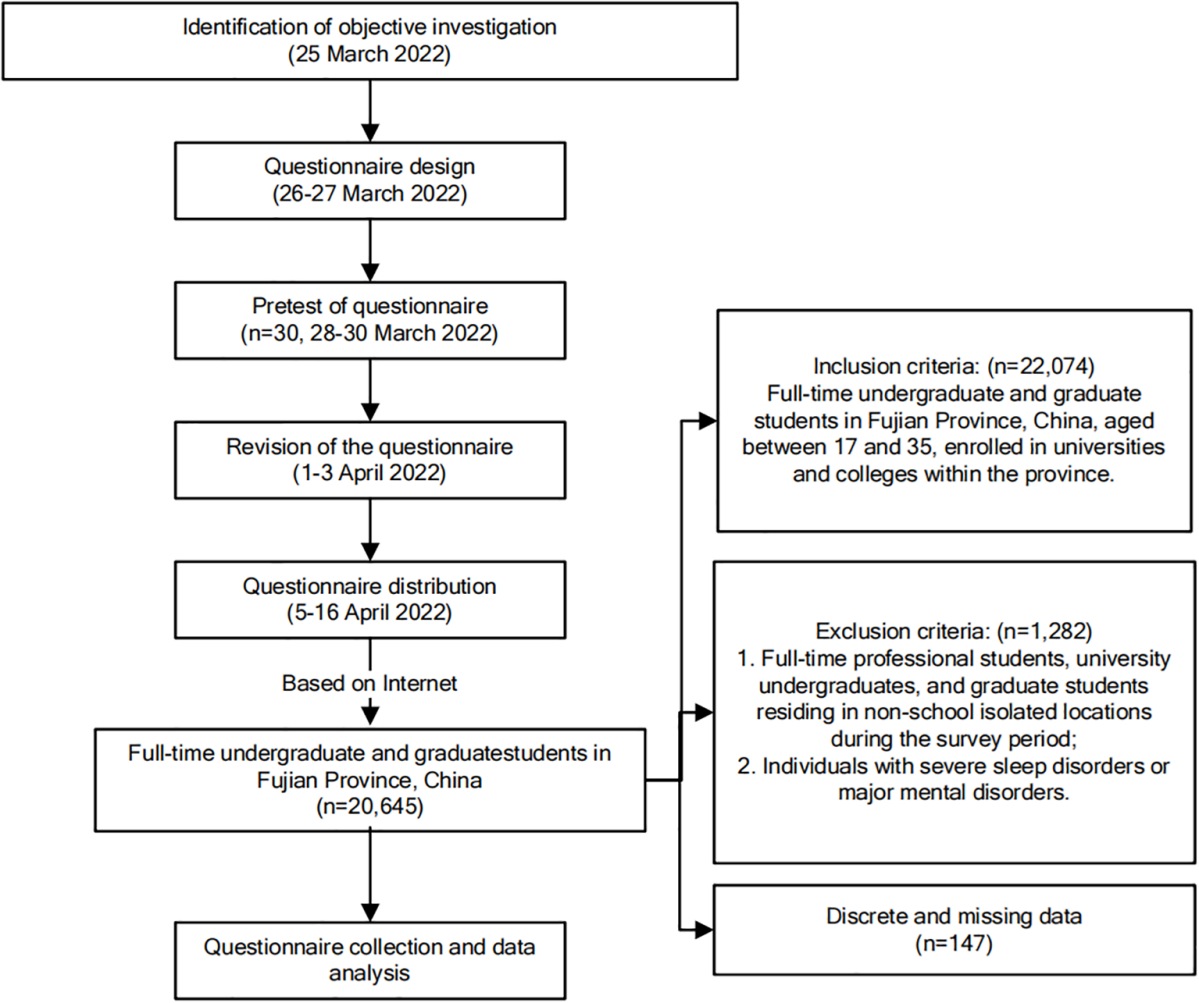
Investigation process.

The ethical review of the study received approval from the University Key Laboratory of Sleep Medicine in Fujian Province and the Sleep Medicine Center of the Second Affiliated Hospital of Fujian Medical University (IRB No. 2021-309). The detailed investigation process is illustrated in **Figure 1**.

### 2.2 Statistical analysis

We employed standard deviations (SD), means, and percentages to delineate continuous and categorical variables from the questionnaires. The independent sample t-test was utilized to compare the seven dimension indexes and PSQI scores between medical and non-medical students.The cut-off value for sleep disturbance is 7. Therefore, we defined PSQI ≤ 7 as good sleep quality and >7 as poor sleep quality at the time of the study (22).

For categorical variables, the chi-square test was employed for analysis. In instances of statistically significant variables in the chi-square test, a multivariate unconditional logistic regression model was applied for further statistical analysis, with results expressed as relative risk odds ratios (OR) and 95% confidence intervals (CI). The questionnaire data were analyzed using the SPSS statistical package (version 25.0) and the R language (version 4.1.0). All p-values are based on two-tailed tests, and values less than 0.05 were considered statistically significant.

### 2.3 Development and validation of prediction models

Prediction models were constructed using the Naive Bayes (NB), Artificial Neural Network (ANN), Decision Tree (DT), and Gradient Boosting Tree (GBT) algorithms. **For feature selection, we chose variables that were statistically significant based on single-factor and multi-factor logistic analyses as predictive features for the different algorithms.** The data were divided into a training set (70%) and a testing set (30%). We employed GridSearch to systematically identify the optimal combination of hyperparameters for the six machine learning algorithms. Meanwhile, to avoid overfitting the accuracy of the model, we used 10-fold cross-validation to evaluate the training and test sets and applied the best model to the testing set. Model performance was assessed by calculating the area under the receiver operating curve (AUROC) of the four models in the testing set. Additionally, we computed accuracy, sensitivity, specificity, precision, and F1-score to further evaluate model performance.

In this study, calibration curve analysis was performed to assess agreement using the slope (ideal value of 1), intercept, and Brier score (ideal value of 0, with values **>**0.3 indicating poor calibration) of the calibration curve. Finally, decision curve analysis was conducted by quantifying the net clinical benefit at different threshold probabilities, and clinical impact curve analysis was performed by quantifying the cost-effectiveness ratio at different threshold probabilities to determine the clinical performance of the prediction model. All machine-learning models were developed and validated using R version 4.2.1.

## 3 Results

### 3.1 Demographic characteristics of participants

This study encompassed a cohort of 20,645 college students from 33 universities in Fujian province. The mean age of participants was (20.70 ± 1.95) years. The majority of students identified as female (n=14,319, 69.4%), and a substantial proportion were non-graduating students (n=19,186, 92.9%). Notably, almost half of the participants refrained from consuming coffee during the survey period (n=10,290, p < 0.05, 49.8%), while a significant number reported staying up late (n=10,951, 53.0%). Approximately 28.9% of students (n=1986) disclosed experiencing poor sleep quality.

In this investigation, college students were stratified into two distinct groups: medical students (4088, 19.8%) and non-medical students (16,557, 80.2%). The comprehensive overview of participant demographics is presented in **Table 1**.

**Table 1:** Characteristics of study participants in this survey (N=20645).

Characteristic	Category	Medical students (4,088)		Non-medical students (16,557)		Univariate analysis	
		Number	Ratio(%)	Number	Ratio(%)	*Chi-square (df)*	*p*
Age	<20	2096	51.3	7131	43.1	88.909 (1)	<0.001
	≥20	1992	48.7	9426	56.9		
Gender	Male	874	21.4	5452	32.9	205.210 (1)	<0.001
	Female	3214	78.6	11105	67.1		
Grade	Graduating class	430	10.5	1029	6.2	91.805 (1)	<0.001
	Non-graduating class	3658	89.5	15528	93.8		
BMI	<18.5	1003	24.5	3801	23	11.716 (3)	0.008
	[18.5,24)	2389	58.4	10124	61.1		
	[24,28)	468	11.4	1835	11.1		
	≥28	228	5.6	797	4.8		
Respiratory history	No	3125	76.4	12352	74.6	5.819 (1)	0.016
	Yes	963	23.6	4205	25.4		
Coffee consumption	No	2312	56.6	7978	48.2	117.350 (3)	<0.001
	Occasionally	1510	36.9	6821	41.2		
	Often	189	4.6	1252	7.6		
	Almost everyday	77	1.9	506	3.1		
Stay up	Not matched	2399	58.7	8552	51.7	88.233 (3)	<0.001
	Sometimes matched	1423	34.8	6315	38.1		
	Often matched	202	4.9	1240	7.5		
	Always matched	64	1.6	450	2.7		
Long hours on the Internet	Not matched	1295	31.7	4597	27.8	44.869 (3)	<0.001
	Sometimes matched	1781	43.6	7134	43.1		
	Often matched	725	17.7	3280	19.8		
	Always matched	287	7	1546	9.3		
Sudden changes	No	3937	96.3	15855	95.8	2.333 (1)	0.127
	Yes	151	3.7	702	4.2		
Fears of infection	Not matched	1431	35	6607	39.9	49.065 (3)	<0.001
	Sometimes matched	2224	54.4	8050	48.6		
	Often matched	329	8	1344	8.1		
	Always matched	104	2.5	556	3.4		
Impatient closed-loop management	Not matched	1505	36.8	6158	37.2	15.626 (3)	0.001
	Sometimes matched	1851	45.3	7093	42.8		
	Often matched	433	10.6	1819	11		
	Always matched	299	7.3	1487	9		
PSQI	≤7	3120	76.3	11553	69.8	67.968 (1)	<0.001
	>7	968	23.7	5004	30.2		

### 3.2 Sleep quality of college students in different majors in Fujian Province during the epidemic

The Pittsburgh Sleep Quality Index (PSQI) scores across seven factors were as follows: 1.063 ± 0.745 for subjective sleep quality, 1.339 ± 0.966 for sleep onset latency, 0.865 ± 0.723 for sleep duration, 0.685 ± 0.848 for sleep efficiency, 1.020 ± 0.626 for sleep disturbances, and 0.07 for the use of sleep medications. Additionally, scores for daytime dysfunction were 0 ± 0.365 and 1.344 ± 0.980. Upon comparing the seven dimensions and PSQI scores between medical students and their non-medical counterparts, a t-test analysis revealed statistically significant differences in five dimensions and overall PSQI scores between the two groups (P<0.05). These findings are meticulously outlined in **Table 2**.

**Table 2:** Comparison of sample sleep quality scores ( $\bar{x}$ ± s).

Dimensionality	Medical students	Non-medical students	*Cohen’s d*	*t*	*p*
Subjective sleep quality	1.015 ± 0.736	1.075 ± 0.746	0.080	-4.647	<0.001
Sleep onset latency	1.259 ± .945	1.359 ± 0.970	0.104	-6.052	<0.001
Sleep duration	0.795 ± 0.724	0.882 ± 0.722	0.120	-6.880	<0.001
Sleep efficiency	0.688 ± 0.830	0.684 ± 0.853	0.005	0.290	0.772
Sleep disturbances	0.960 ± 0.614	1.035 ± 0.628	0.120	-6.870	<0.001
Use of sleep medications	0.058 ± 0.325	0.073 ± 0.374	0.042	-2.640	0.008
Daytime dysfunction	1.122 ± 0.966	1.398 ± 0.976	0.283	-16.314	<0.001
Global PSQI score	5.58 ± 3.066	6.13 ± 3.113	0.145	-10.196	<0.001

### 3.3 Analysis of factors influencing sleep quality among medical students

During the 2022 COVID-19 outbreak in Fujian Province, univariate analysis was employed to investigate the factors affecting the sleep quality of medical students.

A chi-square test revealed a significant correlation between the sleep quality of medical students and eight variables (P < 0.001), which included Grade, Respiratory history, Coffee consumption, Stay up, Long hours on the Internet, Sudden changes, Fear of infection, and Impatient closed-loop management. However, no significant associations were observed with body mass index (BMI), gender, or age.

The Pittsburgh Sleep Quality Index (PSQI) score was selected as the dependent variable, with values of 1 representing poor sleep quality and 0 indicating good sleep quality. This choice facilitated the identification of predictors for the sleep quality of college students during the epidemic. Multivariate unconditional logistic regression analysis was conducted, utilizing the eight statistically significant variables as independent variables to ascertain the predictors of college students’ sleep quality during the epidemic.

The results demonstrated that the non-graduating class was negatively associated with impaired sleep quality, while often coffee consumption, staying up late, long hours spent on the internet, sudden changes, and impatient closed-loop management were positively associated with impaired sleep quality. Further details can be found in **Table 3**.

**Table 3:** Analysis of factors affecting sleep quality [number] among medical students in this survey.

Variables	N=4,088	Sleep Quality		Univariate analysis		Multivariate analysis	*p*
		Good(≤7)	Poor(>7)	*χ^2^*	*p*	OR(95%CI)	
Age							
<20	2096	1625(52.1)	471(48.7)	3.336	0.068		
≥20	1992	1495(47.9)	497(51.3)				
Gender							
Male	874	659(21.1)	215(22.2)	0.458	0.498		
Female	3214	2461(78.9)	753(77.8)				
Grade							
Graduating class	430	295(9.5)	135(13.9)	15.359	<0.001	1.00 [Reference]	
Non-graduating class	3658	2825(90.5)	833(86.1)			0.72 [0.57, 0.91]	0.006
BMI							
<18.5	1003	777(24.9)	226(23.3)	3.205	0.361		
[18.5,24)	2389	1824(58.5)	565(58.4)				
[24,28)	468	343(11.0)	125(12.9)				
≥28	228	176(5.6)	52(5.4)				
Respiratory history							
No	3125	2427(77.8)	698(72.1)	12.927	<0.001	1.00 [Reference]	
Yes	963	693(22.2)	270(27.9)			1.18 [0.99, 1.40]	0.070
Coffee consumption							
No	2312	1838(58.9)	474(49.0)	58.086	<0.001	1.00 [Reference]	
Occasionally	1510	1124(36.0)	386(39.9)			1.08 [0.91, 1.27]	0.370
Often	189	113(3.6)	76(7.9)			1.51 [1.08, 2.11]	0.016
Almost everyday	77	45(1.4)	32(3.3)			1.47 [0.88, 2.43]	0.138
Stay up							
Not matched	2399	1973(63.2)	426(44.0)	149.48	<0.001	1.00 [Reference]	
Sometimes matched	1423	999(32.0)	424(43.8)			1.59 [1.35, 1.89]	<0.001
Often matched	202	122(3.9)	80(8.3)			1.66 [1.19, 2.31]	0.003
Always matched	64	26(0.8)	38(3.9)			2.72 [1.55, 4.83]	0.001
Long hours on the Internet							
Not matched	1295	1116(35.8)	179(18.5)	213.110	<0.001	1.00 [Reference]	
Sometimes matched	1781	1370(43.9)	411(42.5)			1.33 [1.08, 1.64]	0.007
Often matched	725	492(15.8)	233(24.1)			1.78 [1.40, 2.26]	<0.001
Always matched	287	142(4.6)	145(15.0)			3.44 [2.54, 4.67]	<0.001
Sudden changes							
No	3937	3032(97.2)	905(93.5)	27.217	<0.001	1.00 [Reference]	
Yes	151	88(2.8)	63(6.5)			1.99 [1.38, 2.84]	<0.001
Fears of infection							
Not matched	1431	1137(36.4)	294(30.4)	29.434	<0.001	1.00 [Reference]	
Sometimes matched	2224	1692(54.2)	532(55.0)			1.07 [0.90, 1.27]	0.476
Often matched	329	226(7.2)	103(10.6)			1.28 [0.96, 1.70]	0.095
Always matched	104	65(2.1)	39(4.0)			1.38 [0.87, 2.18]	0.165
Impatient closed-loop management							
Not matched	1505	1313(42.1)	192(19.8)	281.910	<0.001	1.00 [Reference]	
Sometimes matched	1851	1398(44.8)	453(46.8)			1.82 [1.50, 2.22]	<0.001
Often matched	433	262(8.4)	171(17.7)			3.26 [2.51, 4.23]	<0.001
Always matched	299	147(4.7)	152(15.7)			4.44 [3.32, 5.95]	<0.001

### 3.4 Analysis of factors influencing sleep quality among non-medical students

Univariate analysis was utilized to investigate the factors influencing the sleep quality of non-medical students. The results of the chi-square test revealed that the sleep quality of non-medical students was associated with nine variables: Age, Gender, Respiratory history, Coffee consumption, Stay up, Long hours on the Internet, Sudden changes, Fear of infection, and Impatient closed-loop management during the local COVID-19 epidemic in Fujian Province in 2022 (P < 0.001). However, this relationship appeared to be independent of body mass index (BMI) and academic grade.

To discern the predictors of sleep quality among non-medical students during the pandemic, we designated the PSQI score as the dependent variable (poor sleep was coded as 1 and good sleep was coded as 0). Subsequently, a multivariate unconditional logistic regression analysis was conducted, with the nine variables showing statistical significance, serving as independent variables to identify predictors of sleep quality among non-medical students during the pandemic.

The findings indicated that a total of nine variables were significantly associated with sleep quality: Age, Gender, Respiratory history, Coffee consumption, Stay up, Long hours on the Internet, Sudden changes, Fear of infection, and Impatient closed-loop management. Detailed results are presented in **Table 4**.

**Table 4:** Analysis of factors affecting sleep quality [number] among non-medical students in this survey.

Variables	N=16,557	Sleep Quality		Univariate analysis		Multivariate analysis	*p*
		Good(≤7)	Poor(>7)	*χ^2^*	*p*	OR(95%CI)	
Age							
<20	7131	5194(45.0)	1937(38.7)	55.353	<0.001	1.00 [Reference]	
≥20	9426	6359(55.0)	3067(61.3)			1.33 [1.24, 1.43]	<0.001
Gender							
Male	5452	4088(35.4)	1364(27.3)	104.040	<0.001	1.00 [Reference]	
Female	11105	7465(64.6)	3640(72.7)			1.26 [1.16, 1.36]	<0.001
Grade							
Graduating class	1029	703(6.1)	326(6.5)	1.034	0.309		
Non-graduating class	15528	10850(93.9)	4678(93.5)				
BMI							
<18.5	3801	2604(22.5)	1197(23.9)	6.389	0.094		
[18.5,24)	10124	7136(61.8)	2988(59.7)				
[24,28)	1835	1261(10.9)	574(11.5)				
≥28	797	552(4.8)	245(4.9)				
Respiratory history							
No	12352	8915(77.2)	3437(68.7)	132.110	<0.001	1.00 [Reference]	
Yes	4205	2638(22.8)	1567(31.3)			1.39 [1.29, 1.50]	<0.001
Coffee consumption							
No	7978	6046(52.3)	1932(38.6)	340.860	<0.001	1.00 [Reference]	
Occasionally	6821	4528(39.2)	2293(45.8)			1.22 [1.13, 1.32]	<0.001
Often	1252	686(5.9)	566(11.3)			1.60 [1.41, 1.83]	<0.001
Almost everyday	506	293(2.5)	213(4.3)			1.31 [1.07, 1.59]	0.008
Stay up							
Not matched	8552	6546(56.7)	2006(40.1)	611.410	<0.001	1.00 [Reference]	
Sometimes matched	6315	4196(36.3)	2119(42.3)			1.33 [1.23, 1.44]	<0.001
Often matched	1240	624(5.4)	616(12.3)			2.02 [1.77, 2.31]	<0.001
Always matched	450	187(1.6)	263(5.3)			2.25 [1.82, 2.78]	<0.001
Long hours on the Internet							
Not matched	4597	3722(32.2)	875(17.5)	761.970	<0.001	1.00 [Reference]	
Sometimes matched	7134	5113(44.3)	2021(40.4)			1.30 [1.18, 1.44]	<0.001
Often matched	3280	1965(17.0)	1315(26.3)			1.89 [1.70, 2.11]	<0.001
Always matched	1546	753(6.5)	793(15.8)			2.64 [2.31, 3.02]	<0.001
Sudden changes							
No	15855	11175(96.7)	4680(93.5)	87.437	<0.001	1.00 [Reference]	
Yes	702	378(3.3)	324(6.5)			1.86 [1.58, 2.19]	<0.001
Fears of infection							
Not matched	6607	4785(41.4)	1822(36.4)	93.382	<0.001	1.00 [Reference]	
Sometimes matched	8050	5610(48.6)	2440(48.8)			0.97 [0.90, 1.05]	
Often matched	1344	829(7.2)	515(10.3)			1.19 [1.04, 1.35]	0.012
Always matched	556	329(2.8)	227(4.5)			1.26 [1.04, 1.53]	0.018
Impatient closed-loop management							
Not matched	6158	4937(42.7)	1221(24.4)	769.310	<0.001	1.00 [Reference]	
Sometimes matched	7093	4854(42.0)	2239(44.7)			1.52 [1.40, 1.66]	<0.001
Often matched	1819	1031(8.9)	788(15.7)			2.19 [1.94, 2.47]	<0.001
Always matched	1487	731(6.3)	756(15.1)			2.76 [2.43, 3.14]	<0.001

### 3.5 Development and validation of a machine-learning-based prediction model for sleep quality in college students across different majors

#### 3.5.1 Predictive feature selection

Based on the results of the multivariate unconditional logistic regression analysis, variables associated with sleep quality were integrated into the prediction model to identify college students from various majors experiencing poor sleep quality (**Tables 3**, **4**).

**Figure 2:**
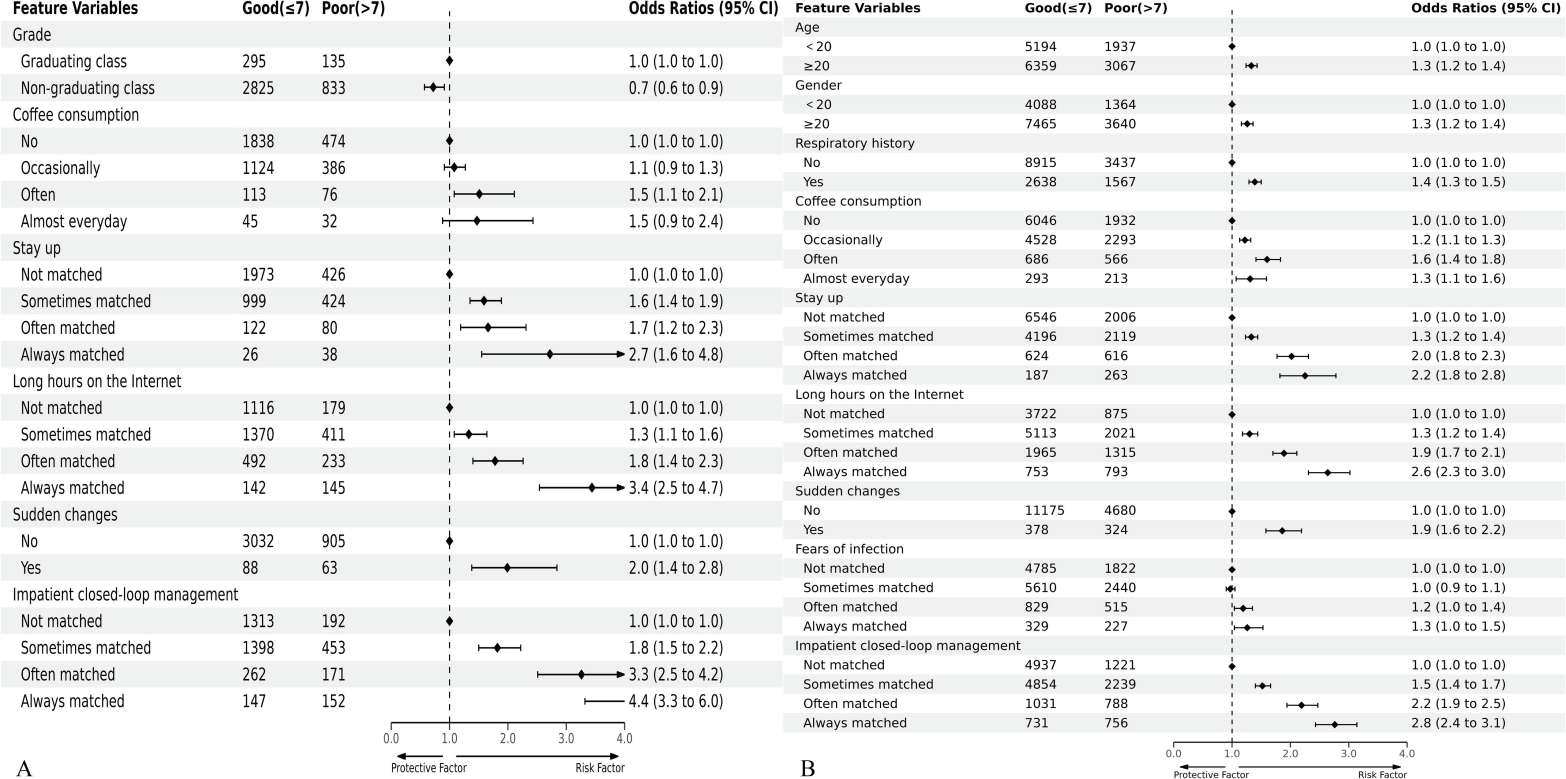
Forest plot of odds ratios (ORs) for predictors included in the prediction model of sleep quality in medical students **(A)** and non-medical students **(B)**. The black points and horizontal lines correspond to the ORs and 95% confidence intervals (CIs). ORs with 95% CIs are indicated on the right in the plot.

In constructing the sleep quality prediction model for medical students, a set of six input variables was employed, comprising Grade, Coffee consumption, Stay up, Long hours on the Internet, Sudden changes, Impatient closed-loop management. The Odds Ratios (OR) and their corresponding 95% Confidence Intervals (CI) for these six predictors are illustrated in **Figure 2A**.

Conversely, the sleep quality prediction model for non-medical students encompassed nine predictors, namely Age, Gender, Respiratory history, Coffee consumption, Stay up, Long hours on the Internet, Sudden changes, Fears of infection, Impatient closed-loop management. The Odds Ratios and 95% CIs for these nine predictors are presented in **Figure 2B**.

#### 3.5.2 Development and validation of predictive models

**Figure 3:**
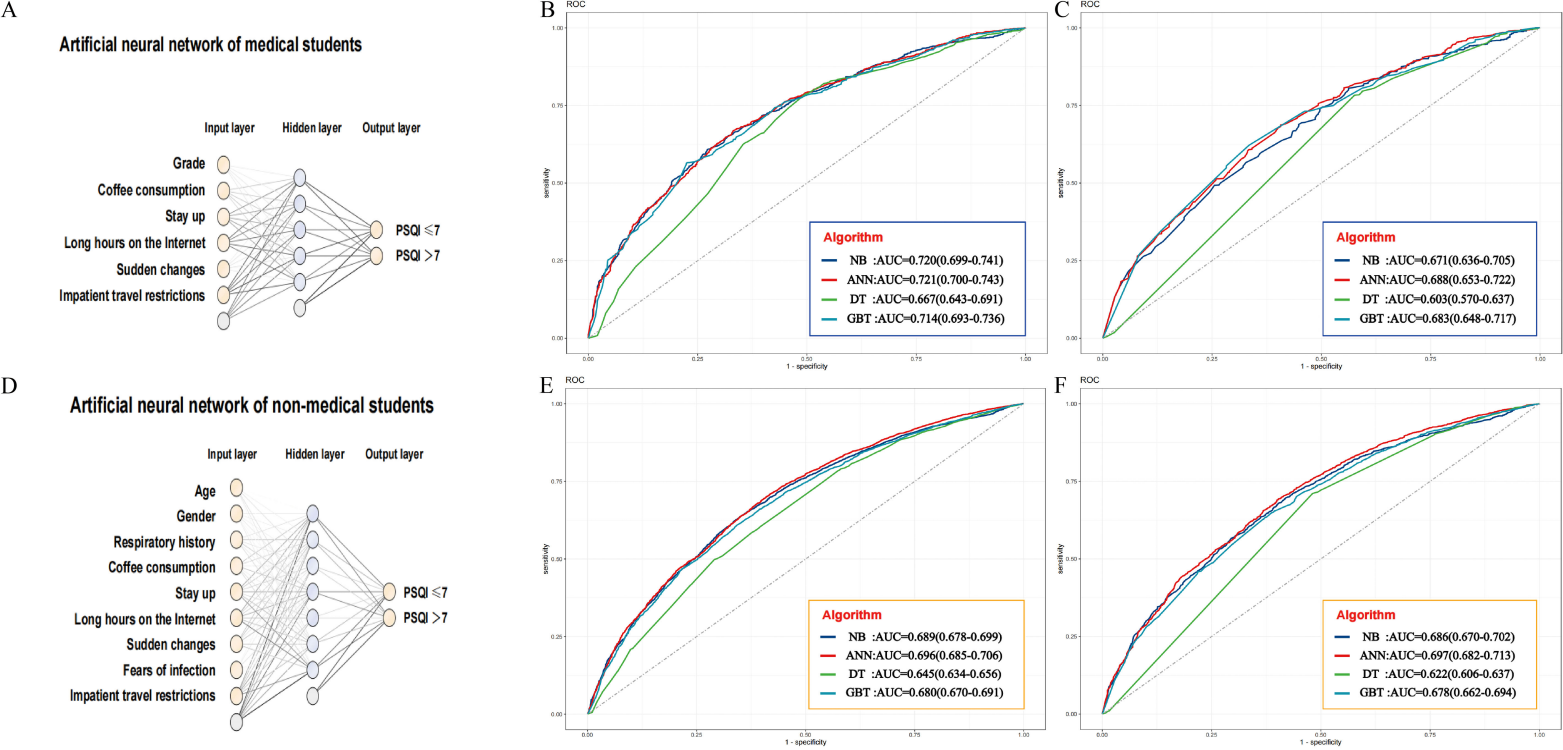
Risk prediction models and ROC curves for sleep quality in medical and non-medical students. **(A)** An artificial neural network architecture for medical students. ROC curves of the training set **(B)** and the test set **(C)** comparing the performance of four algorithms for medical students. **(D)** An artificial neural network architecture for non - medical students. ROC curves of the training set **(E)** and the test set **(F)** comparing the performance of four algorithms for non - medical students.

In the machine learning predictive modeling study with 4,088 medical students, participants were divided into training and testing sets at a 70:30 ratio, with 2,861 (70%) allocated to the training set and 1,227 (30%) to the testing set, ensuring rigorous model development and validation. The six predictors were incorporated into the risk prediction model for sleep quality in medical students (**Figure 3A** and **Supplementary Table S1**). Within the training set, the Area Under the Curve (AUC) values for NB, ANN, DT, and GBT were 0.720, 0.721, 0.667, and 0.714, respectively (**Figure 3B**). In the testing set, the AUCs for NB, ANN, DT, and GBT were 0.671, 0.688, 0.603, and 0.683, respectively (**Figure 3C**). These models exhibited commendable performance, with the ANN model achieving the highest AUC of 0.688. Additionally, we assessed Accuracy, Sensitivity, Specificity, Precision, and F1-score, yielding results of 66.5%, 68.7%, 59.3%, 84.5%, and 75.8%, respectively.

Among the 16,557 non-medical students, 70% (11,590) were assigned to the training set and 30% (4,967) to the testing set for developing a machine learning predictive model. The nine predictors from these participants were amalgamated into a risk prediction model for sleep quality in non-medical students (**Figure 3D**). In the training set, AUC values for NB, ANN, DT, and GBT were 0.689, 0.696, 0.645, and 0.680, respectively (**Figure 3E**). The testing set exhibited AUCs of 0.686, 0.697, 0.622, and 0.679 for NB, ANN, DT, and GBT, respectively (illustrated in **Figure 3F**). Notably, the AUC of the ANN model reached the highest at 0.697, and the Accuracy, Sensitivity, Specificity, Precision, and F1-score were 66.4%, 69.4%, 59.7%, 79.9%, and 74.3%, respectively. Detailed results are provided in **Table 5**.

**Table 5:** Discrimination tests on algorithms for medical students and non-medical students.

Algorithm	Discrimination tests						
	Cutoff	AUROC (95% CI)	Accuracy (95% CI)	Sensitivity (95% CI)	Specificity (95% CI)	Precision (95% CI)	F1-score (95% CI)
Medical students							
NB	0.801	0.671 (0.636-0.705)	0.686 (0.686-0.686)	0.744 (0.772-0.716)	0.500 (0.558-0.442)	0.828 (0.853-0.802)	0.784 (0.810-0.757)
ANN	0.763	0.688 (0.653-0.722)	0.665 (0.665-0.664)	0.687 (0.717-0.657)	0.593 (0.650-0.537)	0.845 (0.871-0.819)	0.758 (0.787-0.729)
DT	0.838	0.603 (0.570-0.637)	0.698 (0.699-0.698)	0.783 (0.810-0.757)	0.424 (0.481-0.367)	0.814 (0.840-0.789)	0.798 (0.825-0.773)
GBT	0.757	0.683 (0.648-0.717)	0.632 (0.633-0.632)	0.622 (0.653-0.591)	0.666 (0.720-0.611)	0.857 (0.883-0.831)	0.721 (0.751-0.691)
Non-medical students							
NB	0.758	0.686 (0.670-0.702)	0.662 (0.662-0.662)	0.697 (0.712-0.682)	0.580 (0.605-0.555)	0.793 (0.808-0.779)	0.742 (0.757-0.727)
ANN	0.686	0.697 (0.682-0.713)	0.664 (0.664-0.664)	0.694 (0.709-0.678)	0.597 (0.622-0.572)	0.799 (0.813-0.785)	0.743 (0.757-0.728)
DT	0.769	0.622 (0.606-0.637)	0.653 (0.653-0.653)	0.711 (0.726-0.696)	0.519 (0.544-0.494)	0.773 (0.788-0.759)	0.741 (0.756-0.726)
GBT	0.699	0.678 (0.662-0.694)	0.638 (0.638-0.638)	0.649 (0.665-0.633)	0.612 (0.637-0.588)	0.794 (0.809-0.780)	0.714 (0.730-0.699)

**Figure 4:**
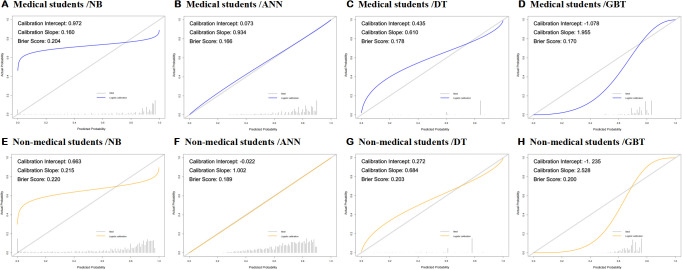
**(A–H)** Calibration curve and Brier score of eight developed models (ANN, Artificial Neural Network; DT, Decision Tree; GBT, Gradient Boosting Tree; NB, Naive Bayes).

Calibration curves for both the prediction model and the ideal scenario were plotted, as depicted in **Figure 4**. Subsequent evaluation focused on the calibration slope, where an ideal value is 1, and the Brier score, with an ideal value of 0 (values > 0.3 indicating poor calibration).

Remarkably, all eight machine-learning models demonstrated good calibration, as illustrated in **Figures 4A–F**. The Brier scores for these models were 0.204, 0.166, 0.178, 0.170, 0.220, and 0.189, respectively. This emphasizes the reliability of our predictive models in approximating the true probabilities of events.

The calibration curves, displayed in **Figure 4**, provide a visual representation of the alignment between predicted probabilities and observed outcomes. The consistency between the predicted and actual values reinforces the robustness of our models in providing accurate predictions.

**Figure 5:**
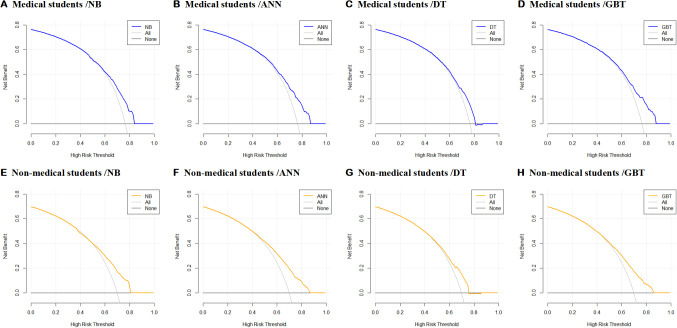
**(A–H)** The decision curve analysis of the model’s clinical decision curve (ANN, Artificial Neural Network; DT, Decision Tree; GBT, Gradient Boosting Tree; NB, Naive Bayes).

To assess the clinical applicability of our prediction model, we conducted decision curve analysis (DCA) and clinical impact curve analysis. The clinical decision curve, illustrated in **Figures 5A–D**, highlights that the NB, ANN, DT, and GBT prediction models exhibit superior net benefits compared to the “treat nothing” or “treat all” strategies. Specifically, in the context of clinical decision-making among medical students, these models demonstrated heightened net benefits at threshold probabilities of 0.84, 0.87, 0.80, and 0.88, respectively.

Interestingly, when applied to non-medical students, the NB, ANN, DT, and GBT prediction models still outperformed the “treat none” or “treat all” strategies. Notably, greater net benefits were observed at threshold probabilities of 0.81, 0.87, 0.75, and 0.86, respectively.

The decision curve analysis of the model’s clinical decision curve, as depicted in **Figure 5**, provides a comprehensive visual representation of the comparative advantages of employing different prediction models in diverse scenarios. This analysis contributes valuable insights into the nuanced effectiveness of these models across varied contexts.

**Figure 6:**
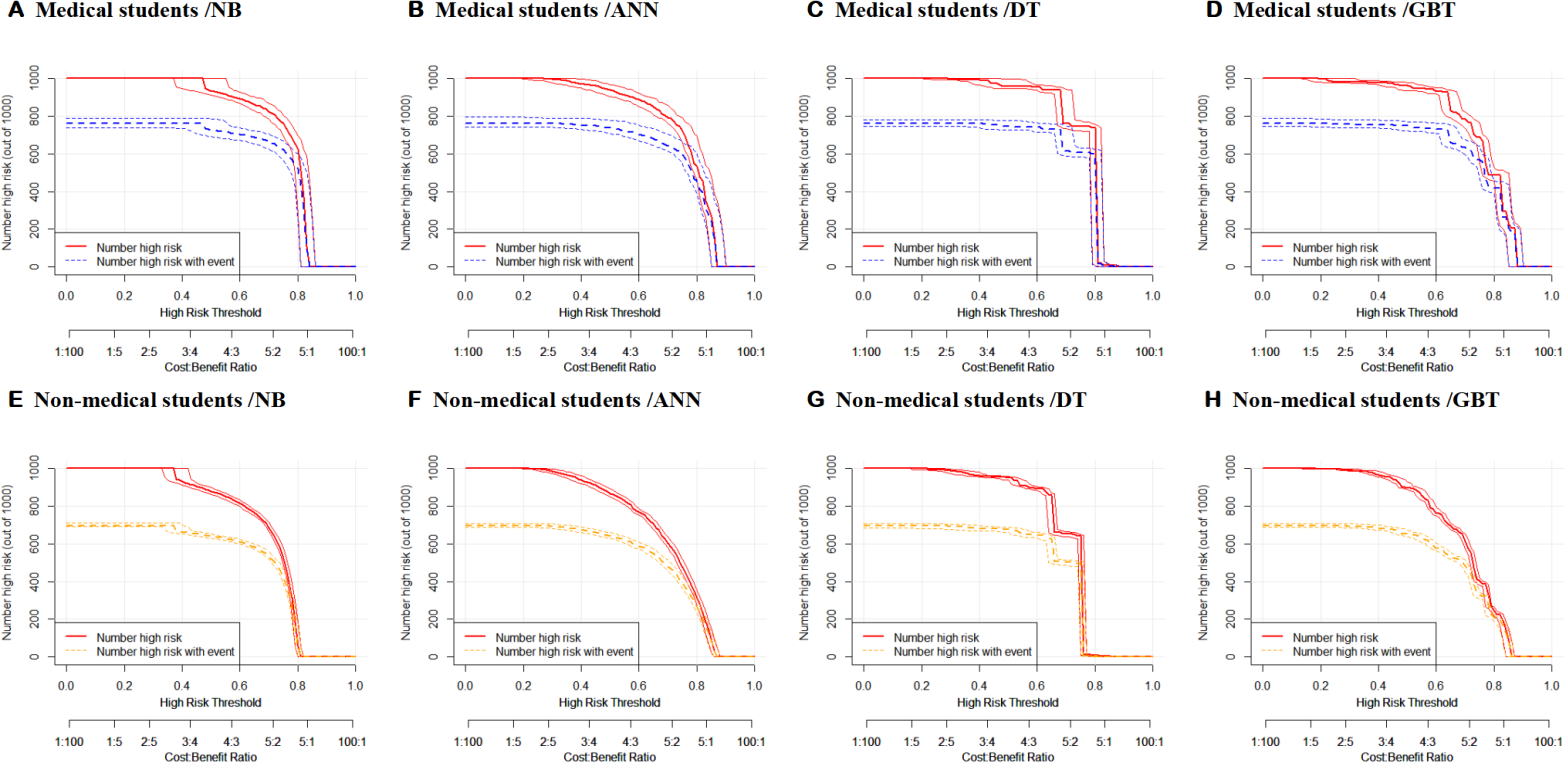
**(A–H)** Clinical impact curve analysis of eight models (ANN, Artificial Neural Network; DT, Decision Tree; GBT, Gradient Boosting Tree; NB, Naive Bayes).

To assess the performance of the eight models in terms of clinical response rate, we conducted clinical impact curve (CIC) analysis. Among medical students, our findings indicate that the NB, ANN, DT, and GBT models exhibit a high degree of accuracy in identifying individuals with poor sleep quality when the threshold probability surpasses 75%, 80%, 70%, and 70%, respectively. These thresholds align closely with the actual instances of poor sleep quality among individuals, as illustrated in **Figures 6A–D**. This outcome robustly validates the efficacy of the prediction model for clinical applications in the context of medical students.

Similarly, in the non-medical student cohort, the NB, ANN, DT, and GBT models demonstrated substantial accuracy in identifying poor sleep quality when the threshold probability exceeded 75%, 75%, 70%, and 70%, respectively. **Figures 6E–H** visually represents the alignment between the models’ assessments and the actual instances of poor sleep quality. This reaffirms the prediction model’s high clinical efficiency, emphasizing its potential utility beyond medical disciplines.

### 3.6 Subgroup analysis of regions with different epidemic severity and the risk of poor sleep quality (PSQI>7)

**Figure 7:**
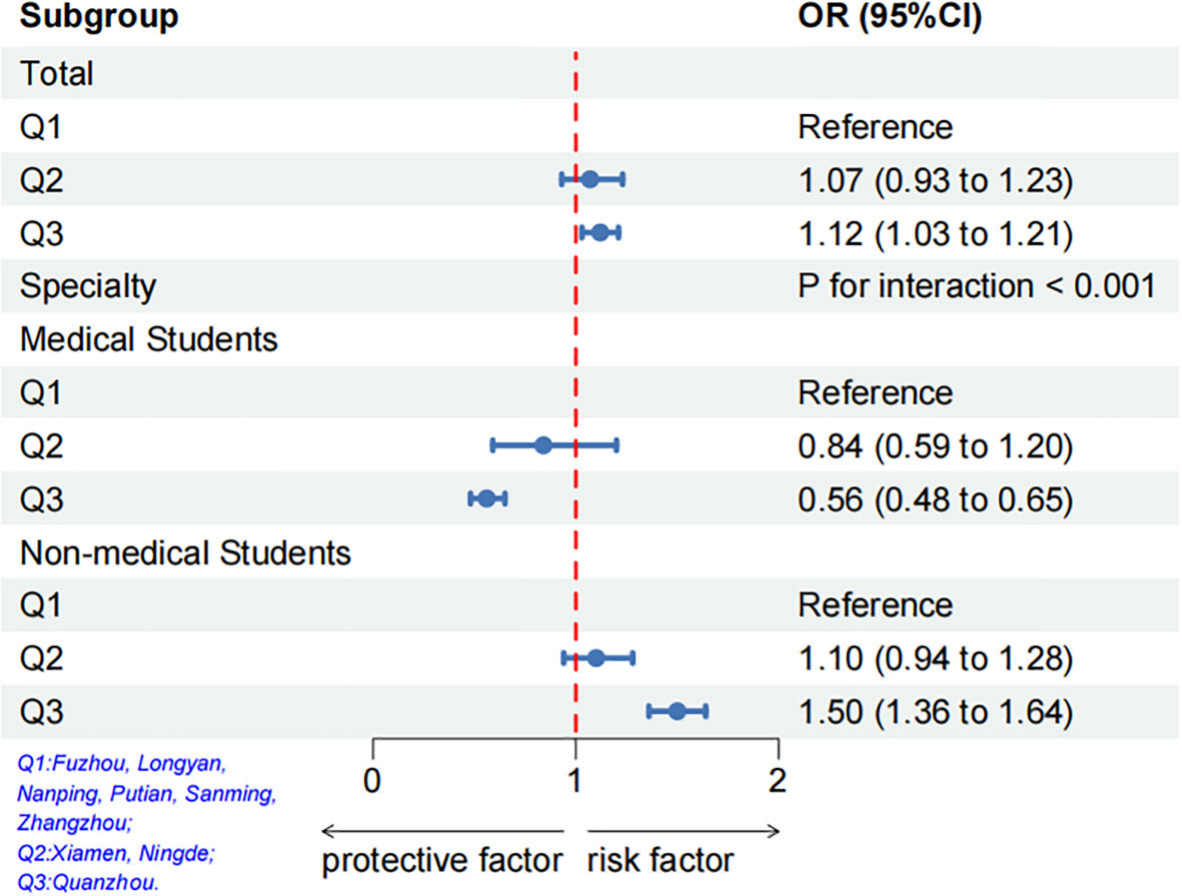
Multivariable logistic regression analysis on the association between the regions with different epidemic severity and the Risk of poor sleep quality after adjusting for confounding factors of age, gender, specialty, grade, and BMI.

Considering the varying severity of the COVID-19 pandemic across different regions of Fujian Province during the pandemic, this study categorized the most severely affected Quanzhou as Q3, followed by Ningde and Xiamen as Q2, while regions without COVID-19 cases such as Fuzhou, Longyan, Nanping, Putian, Sanming, and Zhangzhou were categorized as Q1. This research employed subgroup analysis to further explore the relationship between different levels of pandemic severity and poor sleep quality. As shown in **Figure 7**, as the severity of pandemic increases, it acts as a protective factor for the sleep quality of medical students, while it serves as a risk factor for non-medical students (p for interaction < 0.001).

## 4 Discussion

### 4.1 Analysis of influencing factors of sleep quality

Our study found that Coffee consumption, Staying up, Long hours on the Internet, Sudden changes, and Impatient closed-loop management significantly affected sleep quality in both medical and non-medical students. Pre-graduating medical students had better sleep quality than graduating ones. Liu et al. noted that COVID-19’s impact, fear of leaving campus, competition, employment pressure, and future concerns could cause severe mental health issues in college graduates (23). However, no sleep quality difference was found between graduating and non-graduating non-medical students. This might be due to medical students’ specialized knowledge and future hospital employment, which may increase their pandemic-related anxiety.

We also found factors like age ≥20, female gender, a history of respiratory tract disease, and frequent infection fear to be linked to worse sleep quality. Yet, these factors didn’t significantly affect medical students. Younger non-medical students were more likely to maintain stable employment during the pandemic, which protected their sleep quality. In contrast, older non-medical students faced more employment issues, leading to more worry about pandemic-related societal problems and worse sleep quality.

Previous studies show that female college students have worse sleep quality than male students during COVID-19 (9, 24), though some found no significant gender difference (25). In our non-medical student sample, women were more at risk for sleep disorders than men. The pandemic and quarantine have caused stress to students, with women more distressed than men (26). However, no significant sleep quality gender difference was found among medical students, possibly due to their deeper COVID-19 understanding which may reduce stress impacts.

Non-medical students often worry about infection, leading to increased psychological stress amid rising cases and expanding infected areas, worsening sleep quality (27). Medical students showed no such significant difference. We speculate that after three years of the pandemic, medical students have had more time to access health information and receive medical education, enhancing their self-protection and reducing pandemic sensitivity.

A history of respiratory tract issues also affected non-medical students’ sleep quality. Rhinitis, for example, is linked to sleep disturbances and serves as a risk factor for this group (28). Medical students seemed unaffected, likely due to their better hygiene practices and medical knowledge. They likely integrate health-promoting behaviors into daily routines, mitigating rhinitis’s impact on sleep quality.

Finally, the effect sizes for the differences in PSQI scores between medical and non-medical students were relatively small (Cohen’s d = 0.08 - 0.145). While these differences are statistically significant, we acknowledge that the practical significance of these findings should be carefully considered. The small effect sizes may be due to the complex interplay of factors influencing sleep quality, including psychological stress, lifestyle habits, and environmental conditions. Additionally, the unique challenges faced by medical students during the COVID-19 pandemic, such as increased exposure risk and additional academic pressures, may have contributed to the observed differences.

### 4.2 Development and validation of predictive models

We developed a predictive model to assess sleep quality in medical and non-medical students. For medical students, the model included six variables: Grade, Coffee consumption, Stay up, Long hours on the Internet, Sudden changes, and Impatient closed-loop management. For non-medical students, it included nine variables: Age, Gender, Respiratory history, Coffee consumption, Stay up, Long hours on the Internet, Sudden changes, fear of infection, and Impatient closed-loop management. Unlike prior studies that used variables like anxiety and stress (28), our model focused on accessible parameters. Our model was built using NB, ANN, DT, and GBT methods, showing strong predictive capabilities. ANN performed the best, and calibration curves showed high concordance between predictions and actual results. Decision curve analysis indicated a net benefit, and the clinical impact curve showed good clinical performance.

However, the AUC values of these models in both the training and testing sets were relatively low, with AUC values around 0.7 for medical students and slightly higher for non-medical students. Additionally, the sensitivity and specificity of the models were both below 0.7. These limitations can be attributed to several factors: a) Sample Size: The sample size for medical students was relatively small (approximately 4,000) compared to non-medical students (approximately 16,500). Machine learning models generally require larger datasets to train more robust and accurate models. The smaller sample size for medical students may have led to overfitting in the training set and poorer generalization to the testing set. b) Complexity of Influencing Factors: Sleep quality is influenced by a multitude of factors, including psychological stress, lifestyle habits, and environmental conditions. The models may not have adequately captured the complex interactions between these factors, especially given the unique challenges faced by medical students during the COVID-19 pandemic, such as increased exposure risk and additional academic pressures. c) Data Variability: The data were collected during a specific period of the COVID-19 pandemic, which may have introduced variability and noise that the models were unable to account for effectively. The dynamic nature of the pandemic and the varying levels of stress experienced by students could have contributed to the models’ suboptimal performance.

Despite these limitations, we believe the machine learning models still provide valuable insights into the potential predictors of sleep quality among medical and non-medical students. First, no existing model was found for assessing sleep quality during the COVID-19 pandemic. Secondly, this model offers valuable insights for evaluating sleep patterns and helps administrators and policymakers address sleep-related issues. Finally, it supports the development of targeted interventions to protect students’ well-being amid public health stressors like COVID-19. Misinformation on COVID-19 is widespread online (29), so enhancing laws to curb false information is crucial to reducing student anxiety.

Exercise has immunomodulatory effects and can mitigate depression (30, 31). Educational institutions should promote physical exercise to alleviate psychological burdens and improve sleep quality. Social support networks are critical for enhancing sleep quality (32). Medical students’ sleep quality often surpasses that of non-medical peers due to their professional context. For non-medical students, disseminating accurate public health information can reduce panic. For medical students, optimizing sleep quality can enhance healthcare services. Recommendations include investigating medical students’ mental health, optimizing schedules and syllabi to reduce academic burdens, and improving sleep quality. Integrating prediction models can help identify factors impacting sleep quality and drive improvements. Timely counseling and interventions can promote holistic health. Future research will explore longitudinal study designs like cohort or experimental studies to track sleep quality changes during public health emergencies. Using diverse samples and scenarios will enhance the findings’ generalizability. Investigating long-term time-series data and employing objective data collection methods will strengthen evidence for comparing sleep quality between medical and non-medical students.

### 4.3 Limitations

Our study is subject to several limitations. Firstly, the utilization of a cross-sectional study design precludes the establishment of a causal relationship between the various risk factors and the sleep quality of both medical and non-medical students. As a remedy, future cohort studies are imperative to delve deeper into this complex relationship.

Secondly, our reliance on self-report surveys introduces inherent limitations. This methodology is susceptible to recall bias, subjective feeling interference, and personal expectation bias. These factors may lead to discrepancies between selected response options and the actual circumstances. We have also thought critically about this when designing the questionnaire and analyzing it to ensure that our findings are more convincing. We also plan to use more objective measures of sleep quality, such as actigraphy, to strengthen future studies.

Thirdly, our survey was conducted during the peak of the COVID-19 pandemic in Fujian province and lacked pre- and post-epidemic comparisons. Consequently, the factors influencing sleep quality as reported in our survey may reflect a temporary snapshot.

Fourthly, a notable gap exists as no prior studies have explored the disparities in sleep quality between medical and non-medical students specifically in Fujian province. This limitation hinders our ability to accurately assess the true impact of the COVID-19 pandemic on sleep quality. Moreover, regional variations in the severity of the epidemic within Fujian Province may introduce confounding bias, influencing the robustness of our study’s final results.

Fifthly, our model lacks validation using an external dataset. While our machine learning modeling demonstrates promising predictive power, its reliability is contingent on the development and validation of the utilized data. To enhance the generalizability of our findings, external validation on college students from diverse provinces in China is recommended. External validation on an untouched dataset would also strengthen these models. This step would contribute to the construction of more robust and reliable prediction models.

Sixth, our study is limited to university students in Fujian and may not be representative of students from other regions or countries. Future studies from other regions or countries are needed to validate our results.

Finally, the global PSQI score, when considered as a continuous outcome variable, has limited clinical significance due to the absence of a clear cutoff value. As a result, this study lacks a sensitivity analysis for the binary classification of PSQI scores, which hinders a more detailed examination of the relationship between PSQI scores and their influencing factors.

### 4.4 Future work

To address the limitations identified in this study and further enhance the robustness of the findings, the following directions for future work are proposed:

Expand the Sample Size: Future research should involve a larger and more diverse sample of students, which will help strengthen the generalizability and reliability of the machine learning models.

Incorporate Additional Features: Including a broader range of variables that may affect sleep quality, such as detailed psychological assessments, various lifestyle factors, and environmental conditions, will provide a more comprehensive understanding of the factors influencing sleep.

Explore Advanced Modeling Techniques: Future studies should experiment with more sophisticated machine learning algorithms and techniques, such as ensemble methods and deep learning, to better capture the complex, non-linear relationships between predictors and sleep quality. These advanced models may offer enhanced predictive accuracy and provide deeper insights into the factors that influence sleep patterns during public health crises.

## 5 Conclusions

Medical students in Fujian province reported better sleep quality than non-medical students during COVID-19 outbreaks. Distinct factors affecting sleep quality were found, highlighting the need for tailored interventions for college students during the pandemic. The study developed and validated prediction models, with the ANN model showing high efficiency in predicting sleep quality. University administrators are advised to consider targeted interventions to support student well-being. The study’s findings on sleep quality differences and the ANN model’s success stress the importance of tailored public health measures and predictive tools to address the impact of academic stress and public health crises on college students.

## Data Availability

The raw data supporting the conclusions of this article will be made available by the authors, without undue reservation.
